# Sphingomimetic multiple sclerosis drug FTY720 activates vesicular synaptobrevin and augments neuroendocrine secretion

**DOI:** 10.1038/s41598-017-05948-z

**Published:** 2017-07-20

**Authors:** Frederic D. Darios, Jernej Jorgacevski, Ajda Flašker, Robert Zorec, Virginia García-Martinez, José Villanueva, Luis M. Gutiérrez, Charlotte Leese, Manjot Bal, Elena Nosyreva, Ege T. Kavalali, Bazbek Davletov

**Affiliations:** 1 MRC Laboratory of Molecular Biology, Cambridge, CB2 0QH UK; 2 Laboratory of Neuroendocrinology-Molecular Cell Physiology, Faculty of Medicine, University of Ljubljana, 1000 Ljubljana, Slovenia; 3 Celica Biomedical, 1000 Ljubljana, Slovenia; 4 Institute of Neurosciences, CSIC-Miguel Hernandez University, 03550 Alicante, Spain; 5 Department of Biomedical Sciences, University of Sheffield, Sheffield, S10 2TN UK; 6 Department of Neuroscience, UT Southwestern Medical Center, Dallas, TX 75390 USA; 7 Hôpital Pitié Salpêtrière, ICM, 75013 Paris, France

## Abstract

Neurotransmission and secretion of hormones involve a sequence of protein/lipid interactions with lipid turnover impacting on vesicle trafficking and ultimately fusion of secretory vesicles with the plasma membrane. We previously demonstrated that sphingosine, a sphingolipid metabolite, promotes formation of the SNARE complex required for membrane fusion and also increases the rate of exocytosis in isolated nerve terminals, neuromuscular junctions, neuroendocrine cells and in hippocampal neurons. Recently a fungi-derived sphingosine homologue, FTY720, has been approved for treatment of multiple sclerosis. In its non-phosphorylated form FTY720 accumulates in the central nervous system, reaching high levels which could affect neuronal function. Considering close structural similarity of sphingosine and FTY720 we investigated whether FTY720 has an effect on regulated exocytosis. Our data demonstrate that FTY720 can activate vesicular synaptobrevin for SNARE complex formation and enhance exocytosis in neuroendocrine cells and neurons.

## Introduction

The sphingolipid signalling pathway plays an important role in the brain function and pathophysiology of diverse neurological diseases^1–4^. A synthetic analogue of sphingosine, FTY720 also known as fingolimod, has been investigated extensively for clinical use in the last decade and recently approved for the treatment of multiple sclerosis, the most common inflammatory disorder of the CNS^5, 6^. FTY720, when phosphorylated, binds to sphingosine-1-phosphate receptors and thereby causes lymphocyte egress leading to immunosuppression^7, 8^. Emerging data from experimental animal models suggest that fingolimod may have additional therapeutic applications. Recently, significant effects of FTY720 have been demonstrated in modulation of astrocyte function, neural excitability and anti-cancer effects^9, 10^. FTY720 readily crosses the blood brain barrier and accumulates in the CNS^11, 12^. FTY720 was found to be effective in treatment of animal models of cerebral ischemia, encephalomyelitis, stroke, cerebral malaria and neurodegeneration^12–18^. The observed benefits were accompanied by improvement of electrophysiological abnormalities and synaptic function^19–21^. Recently it has been demonstrated that non-phosphorylated FTY720 can be taken up by neurons increasing long-term potentiation in electrophysiological recordings from hippocampal slices^22^. Given the growing body of *in-vivo* experimentation with FTY720 and its favourable safety profile there is a hope for translation of some of these findings for indications other than multiple sclerosis. Investigation of neuronal mechanisms which could be affected by application of FTY720 thus becomes an important line of research. Since sphingosine was recently shown to act as a stimulator of neuronal exocytosis^23–25^, it is conceivable that FTY720 could also affect release of neurotransmitters and hormones.

Communication between neurons and the release of hormones involve formation of protein complexes between the secretory vesicles and the plasma membrane, where vesicular synaptobrevin engages the plasma membrane syntaxin/SNAP25 heterodimer^26^. Synaptobrevin, resident on synaptic vesicles and chromaffin granules, may not readily react with syntaxin and SNAP-25^27^, although an opposing view has been also put forward^28^. We recently reported that sphingosine, a releasable backbone of sphingolipids, can activate vesicular synaptobrevin for protein assembly and upregulates exocytosis in neuroendocrine cells and in hippocampal neurons^23^. Here we demonstrate that FTY720 is as effective as sphingosine in activating vesicular synaptobrevin and stimulating neuronal and neuroendocrine exocytosis.

## Materials and Methods

### Synaptic vesicle assays and synaptosomal glutamate release

Plasmids encoding glutathione S-transferase (GST) fusion proteins of rat syntaxin 1A (aa 1–261) and rat SNAP-25B were described previously^29^. Recombinant proteins were released from GST by incubating beads with thrombin, and proteins were further purified using a Superdex 200 column equilibrated in 100 mM NaCl, 20 mM HEPES (pH 7.3). Protein concentrations were estimated using Coomassie Plus reagent (Pierce, Thermo Fisher Scientific Inc., Waltham, MA, USA). Synaptosomes were prepared from rat brains on a Ficoll (Pharmacia, Uppsala, Sweden) gradient as described previously^30^. For isolation of synaptic vesicles, synaptosomes were disrupted by the hypoosmotic shock (10 mM Hepes, pH 7.3). Synaptic membranes were removed by centrifugation for 20 min at 34,000 g at 4 °C. Supernatant, containing cytosol and synaptic vesicles, was mixed 1:1 (v/v) with Optiprep (Invitrogen Carlsbad, CA, USA), overlaid with 10 mM HEPES, 100 mM NaCl containing 40% Optiprep, and centrifuged for 1 h at 400,000 g at 4 °C. Synaptic vesicles were collected from the top of the gradient in a 120 µl volume and used in SNARE assembly at 10 µg/reaction. Synaptic vesicles (10 µg) were incubated with premixed syntaxin1/SNAP-25 heterodimers (2 µg) in the presence of 50 µM sphingosine-mimetics (Biomol, Hamburg, Germany) for 30 min at 22 °C. Alternatively, synaptic vesicles (5 µg) were incubated in the presence of Glu-C protease (2.5 ng/µl) for 20 min at 37 °C. Reactions were stopped by the addition of SDS-containing sample buffer. SDS-resistant SNARE complexes were detected by SDS-PAGE followed by Western immunoblotting using an anti-synaptobrevin antibody (clone 69.1, Synaptic Systems, Germany). Anti-synaptophysin antibody was from Synaptic Systems, Germany.

For measurements of glutamate release, freshly-isolated synaptosomes were resuspended in synaptosomal buffer containing (in mM): 132 NaCl, 5 KCl, 20 HEPES, 1.2 NaH_2_PO_4_, 1.3 MgCl_2_, 0.15 Na_2_EGTA, 1 MgSO_4_, 5 NaHCO_3_, 10 D-glucose (pH 7.2). Synaptosomes (1 mg protein/ml) were incubated for 10 min at 37 °C with an equal volume of synaptosomal buffer containing glutamate dehydrogenase (15 units/ml) and 3 mM NADP in the presence of either sphingosine-mimetic solution or vehicle, DMSO. Glutamate release was induced by addition of KCl (35 mM) in the presence of 2 mM CaCl_2_ and monitored by following fluorescence (excitation 340 nm, emission 460 nm) in black 96-well plates in Safire II spectrofluorometer (Tecan, Männedorf, Switzerland). Calcium-dependent release was calculated by subtracting the fluorescence signals obtained in the presence and absence of CaCl_2_.

### Liposomal aggregation assay

Plasmid encoding GST fusion of rat synaptobrevin 2 (aa 1–96) was described previously^29^. Recombinant protein GST-synaptobrevin was eluted from glutathione-Sepharose beads by incubating beads with 10 mM glutathione, and the fusion protein was further purified using a Superdex 200 column equilibrated in 100 mM NaCl, 20 mM HEPES (pH 7.3). Liposomes consisting of phosphatidylcholine/phosphatidylserine (Avanti Polar Lipids) at a molar ratio of 75/25 were prepared by evaporation of chloroform/methanol under nitrogen followed by resuspension in 100 mM NaCl, 20 mM HEPES (pH 7.3) and extrusion through 100 nm filter (Avanti Polar Lipids). 25 µl of GST-synaptobrevin (10 µM) was added to a 75 µl of liposomal solution (0.5 µg/µl) and following 90 sec, 25 µl of solution was added for the final concentration of 0, 10, 20 or 50 µM FTY720. Spectrophotometric changes were recorded at 350 nm using a BioRad spectrophotometer.

### Melanotroph cell experiments

Melanotroph cultures from the pituitary pars intermedia were prepared from male Wistar rats as described previously^31, 32^ and maintained at 37 °C in 5% CO_2_ for up to 4 days. Membrane capacitance (*C*_m_) was measured using the uncompensated whole-cell technique with a dual-phase lock-in patch-clamp amplifier (SWAM IIC, Celica, Ljubljana, Slovenia). Cells were observed under the Zeiss Axio observer A1 (Zeiss, Oberkochen, Germany) in the extracellular solution containing (in mM): 130 NaCl, 5 KCl, 8 CaCl_2_, 1 MgCl_2_, 10 d-glucose, 10 HEPES (pH 7.2). Melanotrophs were voltage clamped at −70 mV and a sine wave voltage (1591 Hz, 1.11 mV RMS) was applied to the pipette (resistance of 2–5 MΩ) which were filled with the intracellular solution containing (in mM): 150 KCl, 2 MgCl_2_, 10 HEPES/KOH (pH 7.2), 2 Na_2_ATP, 2 EGTA, 1.74 CaCl_2_ (yielding free [Ca^2+^] of 1 µM). Signals were acquired by Cell software (Celica, Ljubljana, Slovenia). Secretory responses were measured as a change in *C*_*m*_ (%) relative to the *C*_*m*_ determined immediately after the establishment of the whole-cell recording. We first recorded changes in *C*_*m*_ under spontaneous conditions for 70 s and then added FTY720 (Cayman Chemical, Ann Arbor, MI, USA)-containing solution or vehicle in the ratio (v/v) of 1:1. Changes in *C*_*m*_ were recorded for an additional 70 s.

### Chromaffin cell experiments

Chromaffin cells were isolated from bovine adrenal glands by collagenase digestion and further purified by centrifugation on Percoll gradients as described previously^33^. The cells were maintained at a density of 150,000 cells/cm^2^ in Dulbecco’s modified Eagle’s medium supplemented with 10% fetal calf serum, 10 μM cytosine arabinoside, 10 μM 5-fluoro-2′-deoxyuridine, 50 IU/ml penicillin and 50 μg/ml streptomycin. To study secretion from chromaffin cells, culture media was replaced by Krebs/HEPES, (K/H) basal solution containing (in mM): 134 NaCl, 4.7 KCl, 1.2 KH_2_PO_4_, 1.2 MgCl_2_, 2.5 CaCl_2_, 11 glucose, and 15 HEPES (pH 7.4). Carbon-fiber electrodes with 14 µm diameter tips were used to monitor catecholamine release from individual chromaffin granules (Gil *et al*., 1998). Electrodes were positioned in close apposition to the cell surface using an Axiovert 135 inverted-stage microscope (Zeiss, Oberkochen, Germany) with mounting Hoffman optics (Modulation Optics, Greenvale, NY, USA). An amperometric potential of +650 mV versus an Ag/AgCl bath reference electrode was applied using an Axopatch 200 A amplifier (Axon Instruments Inc. Foster City, CA, USA). Currents generated by catecholamine oxidation were digitized using an A/D converter and recorded using the Clampex program (Axon Instruments Inc. Foster City, CA, USA). Cells were depolarized using 59 mM high potassium-containing K/H solution which was applied through a valve-controlled puffer tip. After acquisition at 2 kHz, individual spike characteristics were analysed using the Quanta program which allows peak detection, integration and parameter calculations^34^. Only well-defined narrow peaks with amplitude higher than 5 pA and stable baseline were used for analysis, ensuring that the secretory events were acquired in the vicinity of the electrodes where maximal catecholamine oxidation is achieved and that multipeaks are discarded from the analysis. The same electrodes were used for the control and FTY-720 treated cells to avoid possible variation.

### Electrophysiology of Rat Hippocampal Neurons

Hippocampal neuronal cultures were prepared from postnatal day 0 to postnatal day 3 Sprague-Dawley rat pups as described^35^. The hippocampus was dissected and dissociated cells were plated on glass coverslips and stored at 37 °C with 5% CO_2_ in a humidified incubator. We used a modified Tyrode’s solution containing (in mM): 145 NaCl, 4 KCl, 2 MgCl_2_, 10 glucose, 10 HEPES, 2 CaCl_2_ (pH 7.4). Pyramidal neurons were whole-cell voltage clamped at −70 mV with borosilicate glass electrodes (3–5 MΩ). Electrode solutions contained (in mM) 105 Cs-methanesulphonate, 10 CsCl, 5 NaCl, 10 HEPES, 20 TEA-Cl, 4 Mg-ATP, 0.3 GTP, 0.6 EGTA, 10 QX-314 (pH 7.3, osmolarity 290 mOsM). Cultured hippocampal cells were visualized with an inverted microscope Zeiss Axiovert S100 (Zeiss, Oberkochen, Germany). Hippocampal cultures (10–21 days *in vitro*) were treated with FTY720 for 10 min at 22 °C, washed thoroughly and then recordings were performed. Recordings were made in whole-cell voltage-clamp mode and cells held at −70 mV. Extracellular solution contained (in mM): 150 NaCl, 4 KCl, 2 MgCl2, 10 glucose, 10 HEPES, and 2 CaCl2, (pH 7.4, 310 mOsm). To isolate AMPA receptor currents induced by EPSCs, recordings were made in the presence of D-AP-5 (50 μM) and picrotoxin (PTX; 50 μM), to block NMDA and GABA-activated currents. The solution also had tetrodotoxin (TTX) (1 μM) in the external solution to suppress action potential firing. FTY720 was dissolved to its final concentration (10 μM) in the extracellular solution. NBQX (10 μM) was used as a specific blocker of AMPA mEPSC. Intracellular solution contained (in mM): 115 Cs-MeSO3, 10 CsCl, 5 NaCl, 10 HEPES, 0.6 EGTA, 20 tetraethylammonium-Cl, 4 Mg-ATP, 0.3 Na2GTP (pH 7.35, 300 mOsm). Currents were recorded with an Axopatch 200 B amplifier and pClamp 9.0 software (Molecular Devices, Sunnyvale, CA, USA). Recordings were filtered at 2 kHz and sampled at 10 kHz. All chemicals were from Sigma Aldrich (St Louis, MO, USA) unless otherwise stated and were of the highest purity available.

### Statistical Analysis

Values are presented as mean ± SEM of the number of experiments indicated in the figure legends or at the top of each bar. Differences between samples were tested with statistical tests indicated in the corresponding figure legends and were considered significant when P < 0.05 (*).

### Ethics Statement

Rats were obtained from Charles River and cared for in accordance with the International Guiding Principles for Biomedical Research Involving Animals developed by the Council for International Organizations of Medical Sciences. All methods were performed in accordance with the relevant guidelines and regulations. Specifically, we have followed the rules of Three R’s to reduce the impact of research on animals. The *ex-vivo* experiments using Wistar rats were approved by the Veterinary Administration of the Republic of Slovenia (Apr. No. 3440-29/2006 and 34401-29/2009/2). The *ex-vivo* experiments using Sprague-Dawley rats were approved by the institutional animal care and use committee (IACUC) protocols of the University of Texas Southwestern Medical Center (APN# 0866-06-05-1).

## Results

**Figure 1:**
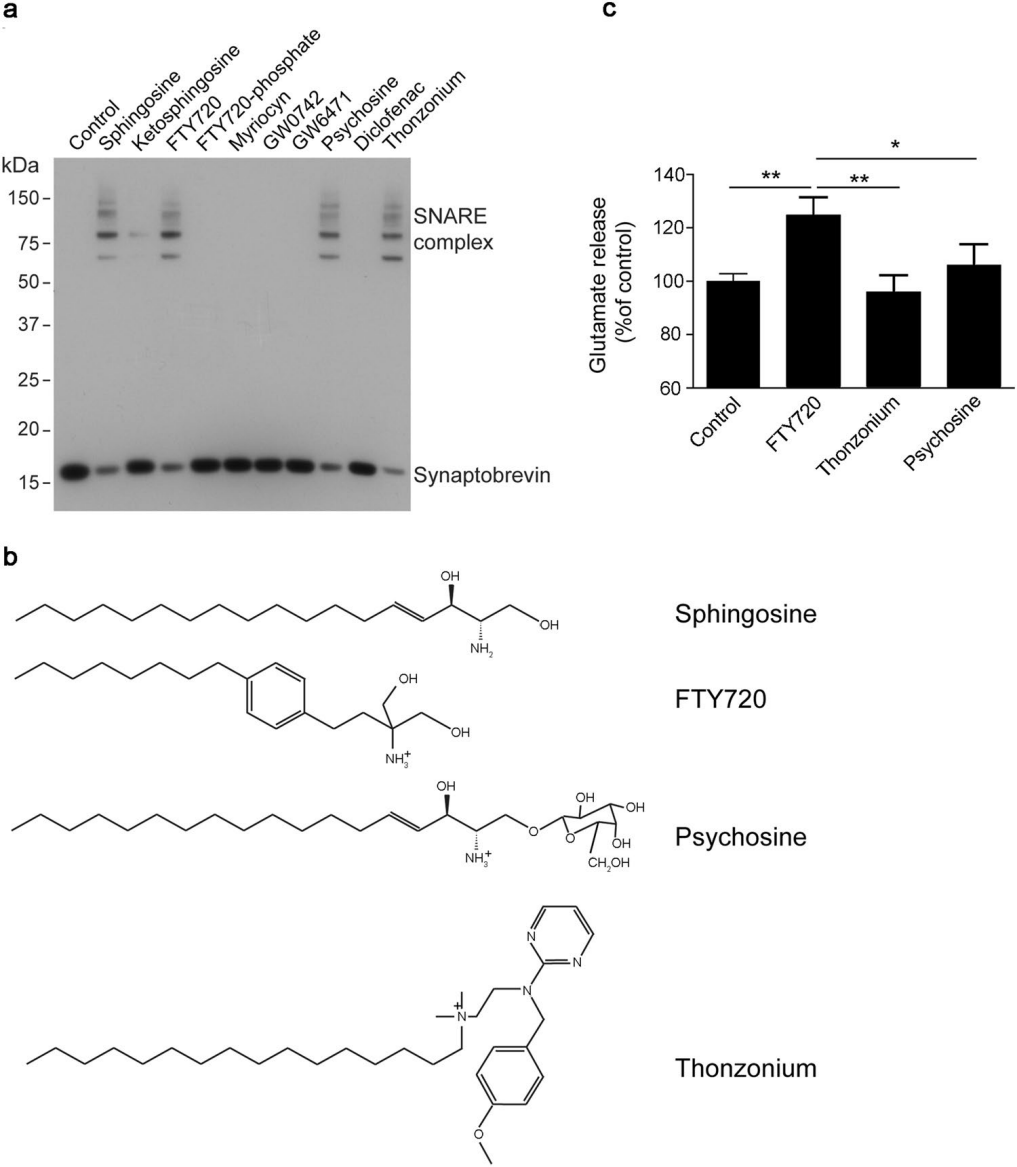
FTY720 induces SNARE complex assembly and potentiates synaptic glutamate release. (**a**) Immunoblot showing that several positively charged compounds (50 μM,﻿ named structures outlined in the panel **b**) can accelerate SNARE complex assembly in the synaptic vesicle assay. (**c**) FTY720 exhibits the strongest positive effect on the glutamate release measured from rat brain synaptic endings, likely due to its superior membrane penetrating properties. All compounds were added at 20 µM 10 min before stimulation of glutamate release with 35 mM KCl (*P < 0.05, **P < 0.01, ANOVA).

In recent years, pharmaceutical companies demonstrated the therapeutic potential of a variety of positively charged lipid compounds including sphingosine mimetics, such as FTY720. We tested whether amine-containing positively-charged lipid compounds could upregulate vesicular synaptobrevin for SNARE complex formation as was reported recently for sphingosine^23^. Synaptic vesicles were purified from rat brain synaptosomes by flotation and then incubated with synaptobrevin’s partner proteins – syntaxin and SNAP25 that normally reside in the plasma membrane. To probe synaptobrevin availability on synaptic vesicles both proteins were prepared recombinantly, without their membrane-interacting parts. When the soluble syntaxin/SNAP25 heterodimer was added to isolated synaptic vesicles, synaptobrevin migrated at its monomeric position as evidenced by Western immunoblotting (control, Fig. 1a), indicating that on the surface of synaptic vesicles synaptobrevin is inhibited. Interestingly, among the tested compounds, only FTY720, psychosine and thonzonium activated vesicular synaptobrevin to engage syntaxin and SNAP25 (Fig. 1a,b). Psychosine (galactosylsphingosine) is a direct derivative of sphingosine and is known to accumulate in the brain tissue in Krabbe’s disease, a rare disorder caused by deficiency of the enzyme galactosylceramidase^36^. Thonzonium is an artificial surface-active agent which is used in topical anti-bacterial medications. FTY720, on the other hand, is prescribed for internal use and was shown to accumulate in the brain^1^. Like sphingosine, FTY720 is positively charged at physiological pH due to its tertiary amine group (pKa 7.8). Importantly, when phosphorylated, FTY720 did not stimulate SNARE assembly (Fig. 1a) suggesting that the positive charge of FTY720 is critical for activation of synaptobrevin.

**Figure 2:**
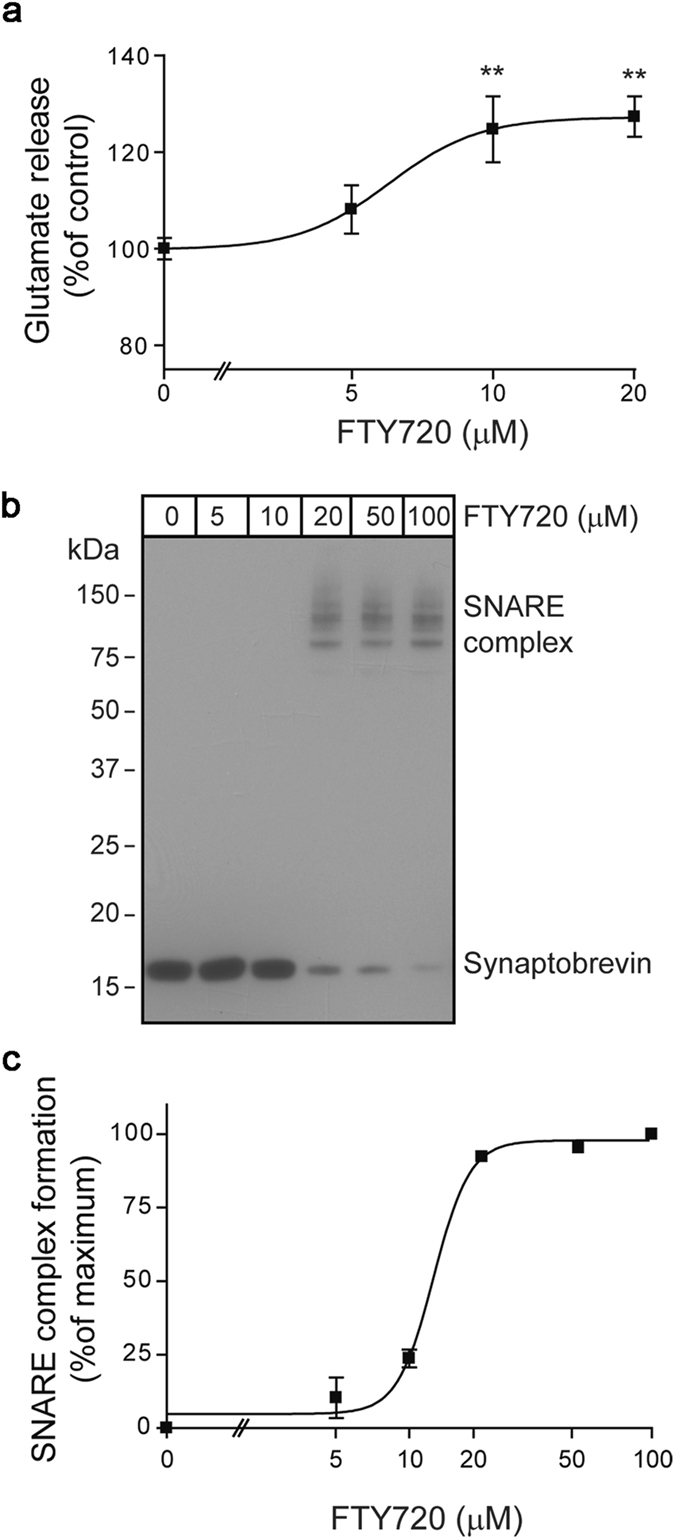
FTY720 accelerates SNARE complex assembly and glutamate release in a dose-dependent manner. (**a**) Graph showing dose-dependence of the FTY720 effect on KCl-triggered glutamate release from rat brain synaptosomes (**P < 0.01, ANOVA). (**b**) Immunoblot showing that synaptobrevin on synaptic vesicles engages syntaxin/SNAP-25 only in the presence of FTY720. (**c**) Graph showing the dose-dependence of the FTY720 effect on SNARE assembly in the synaptic vesicle assay.

Next, we compared the three active compounds on their ability to stimulate calcium-dependent exocytosis from isolated nerve endings obtained from rat brain. The compounds (20 µM) were added to rat brain synaptosomes for 10 minutes before addition of 35 mM KCl to trigger calcium-triggered glutamate release (Fig. 1c). Measure of end-point calcium-dependent release showed that only FTY720 stimulated glutamate release whereas compounds which cannot penetrate lipid membrane due to either a hydrophilic galactose group (psychosine) or a strong positive charge (thonzonium) had no effect. This suggests that FTY720 possesses unique characteristics which are similar to sphingosine and thus we focused further investigation on this particular compound. A titration experiment showed that FTY720 stimulated calcium-dependent glutamate release from rat brain synaptosomes in a dose-dependent manner with EC50 ~7 µM (Fig. 2a). In the SNARE assembly reaction, we observed similar but slightly lower sensitivity of synaptobrevin to FTY720 when using the isolated vesicle preparation (EC50 ~14 µM, Fig. 2b,c).

**Figure 3:**
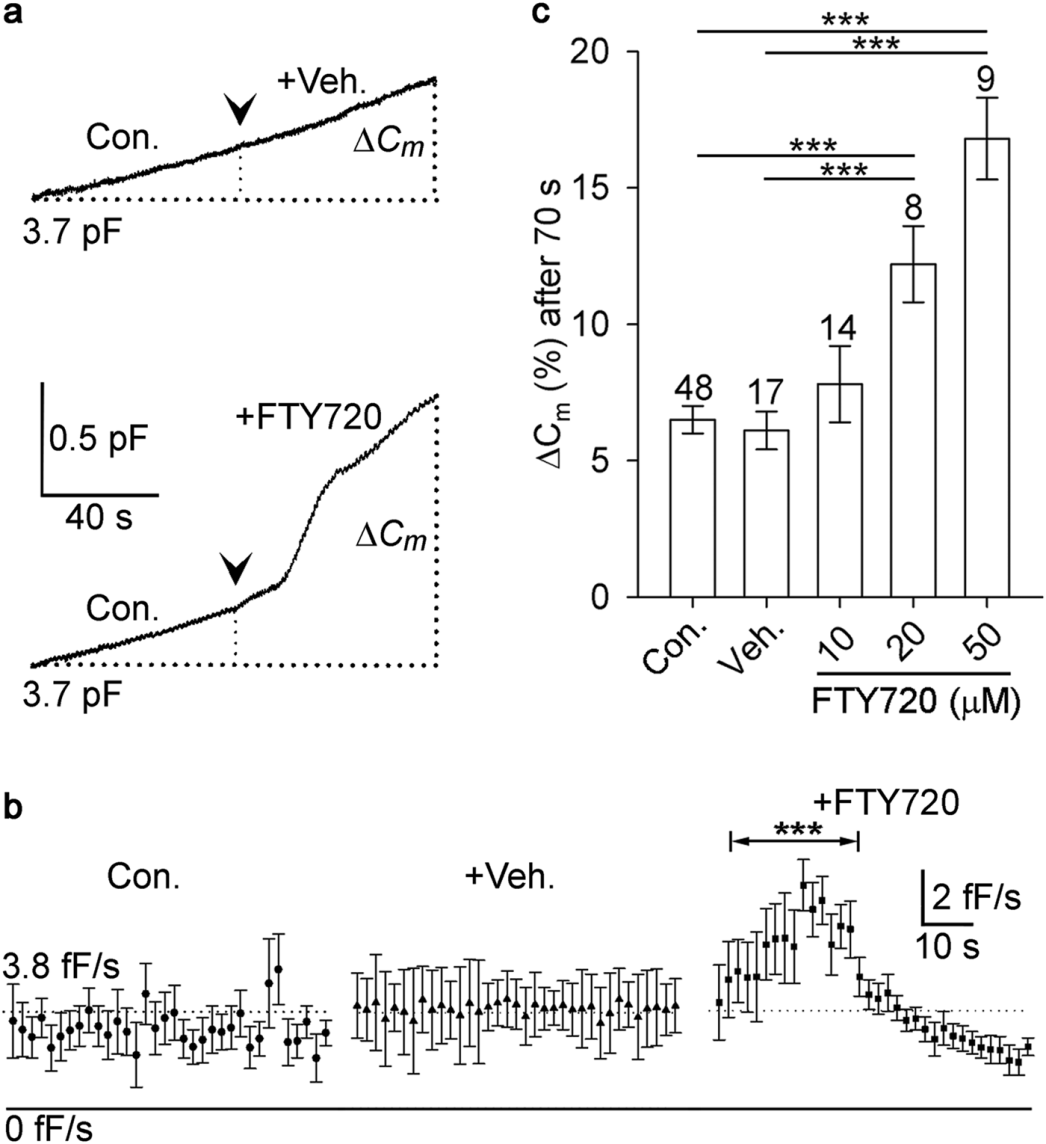
FTY720 enhances stimulated exocytosis in pituitary melanotrophs. (**a**) Representative traces of time-dependent changes in membrane capacitance (*C*_*m*_), measured in control conditions (Con.) and after the addition of either vehicle (+Veh.) or FTY720 (50 μM). Whole-cell Cm measurements were performed in the presence of 1 μM calcium in the patch pipette. (**b**) The averaged changes in the derivative of *C*_*m*_ (*C*_*m*_/dt) in control (Con.), vehicle-treated (+Veh.) and FTY720-treated (+FTY720) conditions. Note the transient increase of the *C*_*m*_ derivative following the addition of FTY720, which indicates enhanced rate of exocytosis (**c**), FTY720 elicited a dose-dependent increase of *C*_*m*_, measured 70 s after the addition of FTY720, at 10 μM, 20 μM and 50 μM concentration (***P < 0.001, Mann–Whitney U-test).

Next we investigated whether FTY720 can upregulate exocytosis in cultured melanotroph neuroendocrine cells isolated from the rat pituitary pars intermedia. We measured changes in the total plasma membrane area by the whole-cell membrane capacitance (*C*_m_) technique, which reflects the balance of exocytosis and endocytosis^37^. Secretory responses were measured for 70 s, relative to the *C*_m_, determined immediately after the establishment of the whole-cell recording (control conditions), or following the addition of either vehicle or FTY720 (Fig. 3a). A steady increase in *C*_m_, due to 1 µM free calcium in the patch pipette solution, was unchanged after the addition of vehicle. In contrast, addition of FTY720 resulted in a pronounced increase in *C*_m_, followed by a decline in *C*_m_, most likely reflecting the activation of compensatory endocytosis (Fig. 3a,b). Dialyzed FTY720 at 10 µM, 20 µM and 50 µM concentrations enhanced the secretory response in melanotrophs by ~23%, ~85% and ~162%, respectively (Fig. 3c). Considering the average diameter of secretory vesicles in melanotrophs being ~140 nm^38^, the acute application of 50 μM FTY720 in the presence of 1 µM [Ca^2+^]_i_ led to fusion of approximately 1300 vesicles with the plasma membrane in 70 s in a single cell, whereas 1 µM [Ca^2+^]_i_ by itself triggered fusion of only ~500 vesicles in the same period of time.

**Figure 4:**
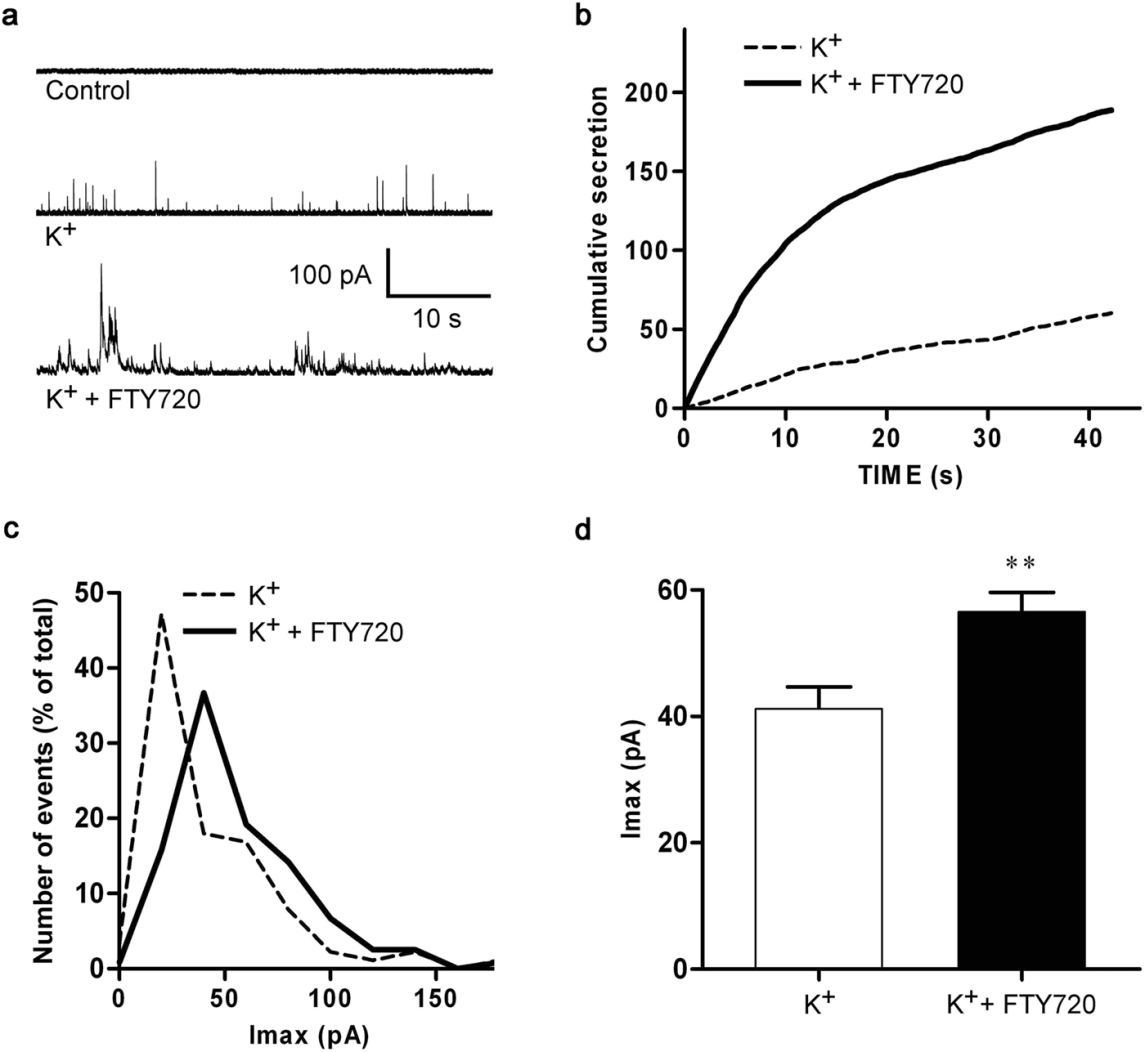
FTY720 enhances chromaffin cell exocytosis stimulated by 59 mM KCl. (**a**) Representative amperometric recordings obtained from cells stimulated by depolarization with a high potassium solution (K^+^) when pre-incubated or not with a 20 µM FTY720 solution for 15 min. (**b**) A graph showing the cumulative secretion obtained by integration of amperometric responses from control (n = 89) and FTY720-treated cells (n = 129). Averaged individual spikes in the form of amplitude distributions (**c**) and the mean amplitude ± SEM (**d**) demonstrate the effect of FTY720 on individual secretory events (**P < 0.005, Student’s t-test).

Fusion of vesicles with the plasma membrane may not always result in the extracellular release of signaling molecules^39^. For instance, the neck connecting the fusing vesicle with the plasma membrane (i.e. the fusion pore) may be too narrow to allow the release of signaling molecules from vesicles^40^. It is therefore important that *C*_m_ measurements are complemented with techniques that report the amount of secreted compound. To assess possible effects of FTY720 on the release of catecholamines from bovine chromaffin cells, we performed amperometric measurements, which allow characterisation of individual fusion events^41^. Catecholamine release from chromaffin cells, pre-treated with FTY720 or not (controls), was stimulated by depolarization with high potassium solution (59 mM KCl). Representative amperometric traces obtained from control as well as FTY720-treated cells are shown in Fig. 4a. Averaging the integrated responses, obtained from 80 cells, demonstrated three-fold enhancement of single vesicle secretion by FTY720 (Fig. 4b). Analysis of individual fusion events revealed transition to higher amplitude values with a 30% increase in the amplitude of the spikes and a two-fold increase in the amount of catecholamines released per event when compared to controls (1.9 × 10^6^ vs 3.8 × 10^6^ catecholamine molecules released, per event, in control and FTY720-treated cells, respectively) (Fig. 4c,d).

**Figure 5:**
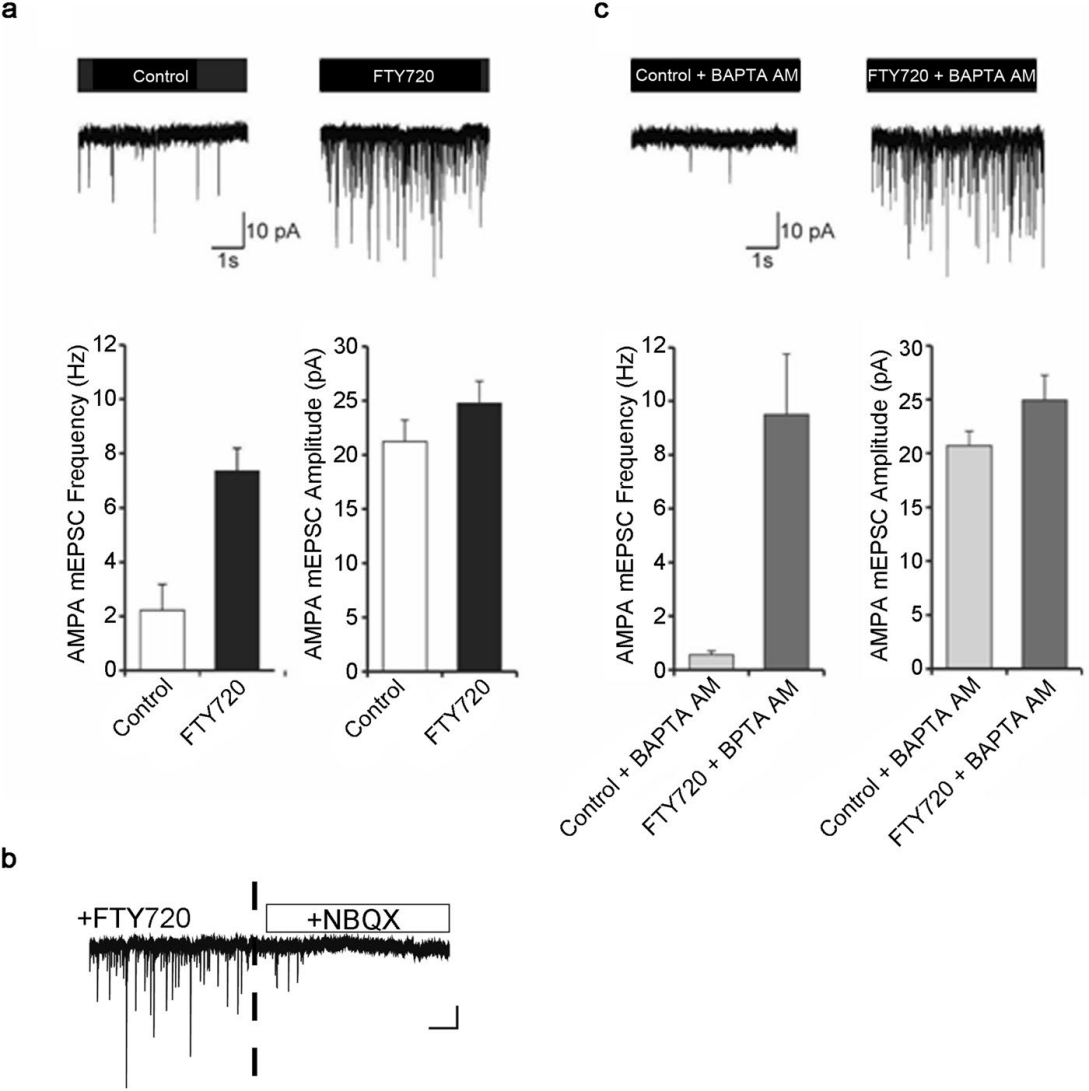
FTY720 augments spontaneous mEPSC activity in rat hippocampal neuronal cultures. (**a**) Representative traces of mEPSCs for control neurons and neurons treated with 10 µM FTY720. A three-fold increase in synaptic mEPSC frequency was detected after application of FTY720 (p < 0.0001, t-test), n = 6 for control, n = 8 for FTY720. Only a small insignificant increase in mEPSC amplitudes was observed after FTY720 application (control 21.2 ± 2; FTY720 24.8 ± 2). (**b**) The stimulating effect of FTY720 can be blocked by 10 µM NBQX, a specific antagonist of AMPA receptors. (**c**) Cell-permeable calcium chelator, BAPTA-AM does not block the stimulating effect of FTY720 on neuronal mEPSC frequency but reduces basal mEPSCs frequency. Application of FTY720 significantly increased mEPSC frequency in neurons pre-treated with BAPTA-AM (p < 0.0001, t-test), n = 5 for control, n = 9 for FTY720. 20% increase in mEPSC amplitudes was observed in the presence of BAPTA-AM (control 20.7 ± 1; FTY720 24.9 ± 2).

The above experiments demonstrated that FTY720 can potentiate stimulated exocytosis. Our next goal was to learn if FTY720 could modulate the spontaneous release of neurotransmitters in the absence of stimulation. Therefore, we measured mEPSC activity in rat hippocampal neurons in culture. The application of FTY720 at 10 µM caused ~3 fold increase in frequency of mEPSCs over the controls, as evidenced by representative traces of mEPSCs (Fig. 5a). FTY720 also slightly increased the mean amplitude of the unitary events by 6-8 pA but this increase was not significant (Fig. 5a). Importantly, the increase in mEPSC frequency could be suppressed by 10 µM NBQX, a blocker of AMPA receptors, indicating the specific nature of FTY720 action (Fig. 5b). To examine whether the observed effects involve calcium-dependent mechanisms, we analysed the action of FTY720 on the mEPSC activity after incubation with BAPTA-AM, a cell-permeable calcium chelator. Neurons were pre-treated with 10 µM BAPTA-AM for 30 min and then mEPSCs were recorded. Figure 5c shows that although BAPTA-AM treatment caused a ~4-fold reduction in baseline mEPSC frequency, under the same conditions FTY720 application still induced strong increase in the frequency of mEPSCs and a slight augmentation of the mean amplitude of the unitary events.

**Figure 6:**
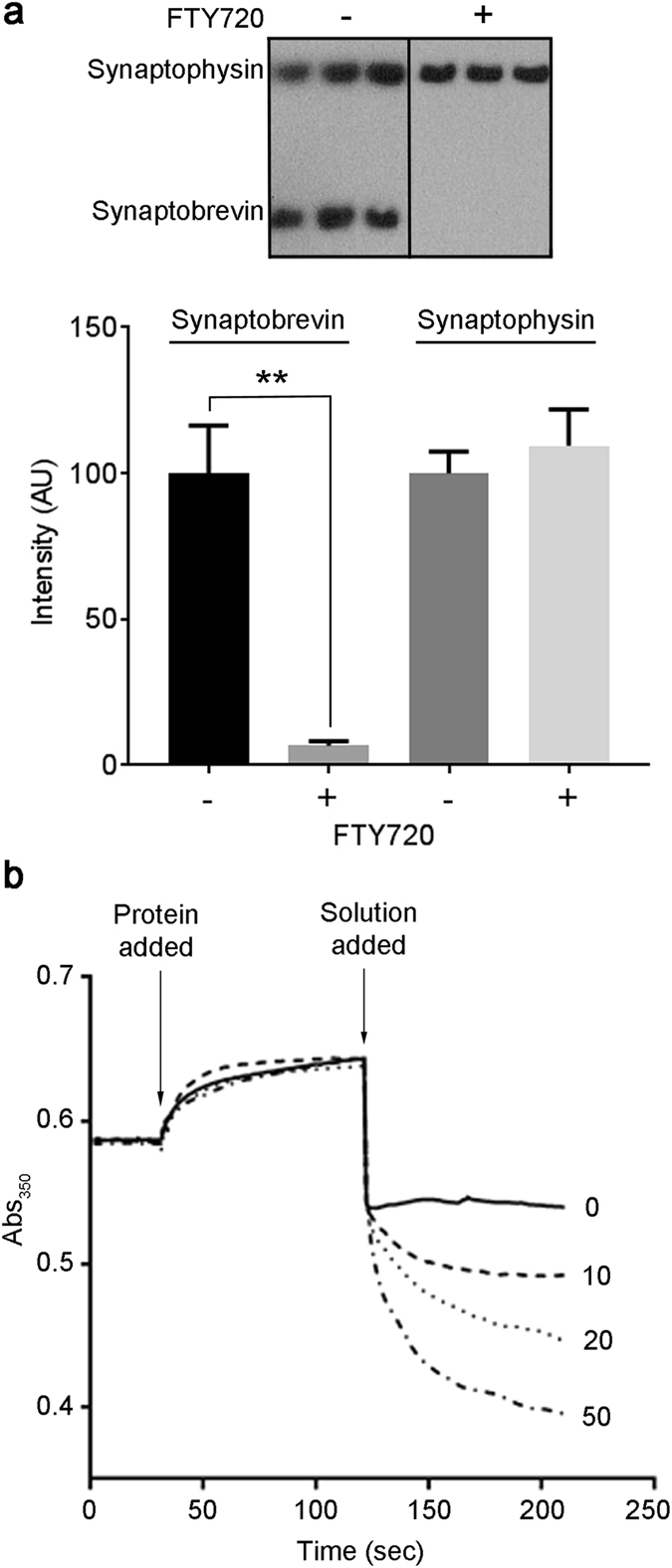
FTY720 disrupts the interaction of the cytosolic part of synaptobrevin with phospholipid membranes. (**a**) Immunoblot showing that native synaptobrevin in rat brain synaptic vesicles (5 µg protein) exhibits faster degradation by GluC protease (2.5 ng/µl) in the presence of FTY720 (50 µM). Synaptophysin immunostaining indicates no change in its proteolytic degradation suggesting a specific action of FTY720 on synaptobrevin (**P < 0.005, ANOVA, n = 3). (**b**) Graph showing that addition of dimeric GST-synaptobrevin (aa 1–96) causes aggregation of phospholipid liposomes as evidenced by a gradual increase of absorbance at 350 nm (Abs_350_). The aggregated state can be reversed by the addition of FTY720 solution at the indicated concentrations. In the absence of FTY720, only a drop due to the dilution factor is observed.

Next we addressed the behaviour of vesicular synaptobrevin in response to FTY720 in a more mechanistic detail. We used a limited proteolysis approach to test whether FTY720 increases availability of synaptobrevin to a proteolytic enzyme. We incubated purified synaptic vesicles in the presence of Glu-C protease, which hydrolyses proteins at glutamine residues, and estimated residual protein content by immunoblotting. Figure 6a shows that Glu-C enzyme was able to digest synaptobrevin more efficiently in the presence of FTY720 (50 µM). In contrast, synaptophysin amounts were not affected by the drug. Thus, FTY720 not only affects synaptobrevin’s availability for SNARE interactions but appears to influence it more generally. Next, we analysed whether synaptobrevin’s cytoplasmic part (aa 1–96) can interact with phospholipid membranes and whether FTY720 can disrupt such an interaction. We prepared liposomes containing phosphatidylcholine (75%) and phosphatidylserine (25%) and incubated them with a fusion protein consisting of glutathione-S-transferase (GST) and synaptobrevin (aa 1–96). Since GST is a dimeric protein, binding of liposomes by synaptobrevin (aa 1–96) would cause liposomal cross-linking which can be evidenced by an increase in absorbance^42^. Figure 6b shows that the addition of GST-synaptobrevin (aa 1–96) caused liposomal aggregation. Crucially, addition of FTY720 led to a dispersal of liposomal aggregates in a dose-dependent manner as evidenced by a gradual decrease in spectrophotometric signal, following the expected drop due to a dilution factor. Addition of the vehicle solution in a control reaction led only to the dilution-related drop. Thus, synaptobrevin’s cytoplasmic part not only interacts with phospholipids but this interaction can also be influenced by lipophilic factors, such as FTY720.

## Discussion

Sphingomimetic FTY720 has been recently approved for treatment of multiple sclerosis, albeit with some side effects which include headaches, fatigue and gastrointestinal disturbance^43, 44^. FTY720 exhibits strong immunosuppressive effects and when phosphorylated it drives sequestration of circulating mature lymphocytes into the lymph nodes^6^. The diverse therapeutic potential of FTY720 may involve new, yet unrecognised effects of FTY720 directly within the CNS. FTY720 readily penetrates into the CNS and accumulates in the brain and in the spinal cord^12^. The need for well-defined therapeutic targets for FTY720 and related drugs has been highlighted^8^. Recent studies demonstrated the role of FTY720 on specific functions of astrocytes such as cytoskeleton^10, 45^, vesicle traffic and calcium homeostasis^9, 46^. In PC12 neuronal model cells, micromolar concentrations of FTY720 activated intracellular protein phosphatase 2 A (PP2A), an important modulator of neuronal function^47^. FTY720 was shown to rescue the functional deficits of synapses in an animal model of multiple sclerosis, suggesting that it can have a direct action in CNS^21^. In the current study, we identify a new target of FTY720 related to synaptic function, namely vesicular synaptobrevin, which plays a crucial role in the release of neurotransmitters and hormones^48^.

The synaptic vesicle assay for SNARE complex assembly^23^ confirmed FTY720 as a positive regulator of synaptobrevin almost identically to sphingosine. Several complementary approaches measuring exocytosis demonstrated that, in a micromolar range, FTY720 has a strong potentiating effect on secretion in several neurosecretory cell models. This enhancement in secretion seems to be in agreement with the effects reported for sphingosine in a variety of cellular systems such as hippocampal neurons and pituitary melanotrophs^23^, lactotrophs^24^, and adrenal chromaffin cells^25, 49^. The structural analogy of FTY720 with sphingosine could underlie the observed recruitment of synaptobrevin for SNARE complex assembly thereby enhancing regulated exocytosis. Although our data do not rule out further effects of FTY720 which could contribute to enhanced exocytosis^9^, we would like to propose that this sphingomimetic drug can also uplift the cytoplasmic part of synaptobrevin thereby activating SNARE assembly for vesicle exocytosis. We do not necessarily envisage that FTY720 directly binds to synaptobrevin – it is possible that after incorporation into the synaptic vesicle membrane the positively charged amphiphilic FTY720 changes the interface between synaptobrevin and the negatively charged surface of the vesicular membrane. The stimulatory effect of FTY720 on synaptobrevin was observed when the drug reaches micromolar range. Pertinently, a study of intracellular accumulation of FTY720 demonstrated that micromolar levels are easily achievable^50^. Using LC-MS mass-spectrometry method the authors demonstrated that the lipophilic FTY720 accumulated several hundredfold inside cells reaching micromolar concentrations. Such cellular accumulation may also explain slightly higher sensitivity of synaptosomal release to FTY720 when compared to the *in vitro* vesicular SNARE complex formation (Fig. 2). It is important to consider that FTY720 is administered internally at mg doses and thus micromolar concentrations can be reached locally simply following drug dissolving in a volume equivalent to a glass of water (1 mg/200 ml equates ~15 µM).

Interestingly, our data revealed that FTY720 not only increased the frequency of secretory events, but also augmented the amount of neurotransmitters released in individual fusion events. Such increase in the spike amplitudes was previously reported for sphingosine^24, 49^ emphasizing a similar effect of positively charged amphiphilic molecules on the single vesicle fusion events in neuroendocrine cells. It is also possible that the small increase in mEPSC amplitudes observed after FTY720 treatment in hippocampal neurons reflects a postsynaptic role for synaptobrevin in AMPA receptor trafficking. Specifically, the increased amplitude of mEPSCs upon FTY720 action could be caused by an increase in the number of glutamate receptors on postsynaptic densities leading to increased amplitude of mEPSCs. Recent experiments showed that postsynaptic AMPA receptor insertion requires synaptobrevin-dependent fusion of receptor-laden vesicles with the postsynaptic plasma membrane^51, 52^.

Together, our data reveal FTY720-triggered enhancement in vesicular fusion in neurons and neuroendocrine cells, urging further investigations of lipid regulators as novel therapeutics for neurological disease, especially because lipid-based drugs readily cross the blood-brain barrier and could accumulate in the brain tissue. FTY720 has been proposed to have potential therapeutic applications in models of Alzheimer’s disease, in cerebral malaria, or cerebral ischemia^11^. Our data therefore provide novel insights into the intracellular actions of FTY720 and may contribute to better understanding of the beneficial action of FTY720 and its side-effects in a variety of neurological conditions. More investigations will need to be carried out to determine the exact binding mechanism by which FTY720 influences synaptobrevin’s interaction with phospholipid membranes and whether it affects other synaptic players and neuronal processes.

## References

[1] Brunkhorst R, Vutukuri R, Pfeilschifter W (2014). Fingolimod for the treatment of neurological diseases-state of play and future perspectives. Front Cell Neurosci.

[2] Davletov B, Montecucco C (2010). Lipid function at synapses. Curr Opin Neurobiol.

[3] Hannun YA, Obeid LM (2008). Principles of bioactive lipid signalling: lessons from sphingolipids. Nat Rev Mol Cell Biol.

[4] He X, Huang Y, Li B, Gong CX, Schuchman EH (2010). Deregulation of sphingolipid metabolism in Alzheimer’s disease. Neurobiol Aging.

[5] O’Connor P (2009). Oral fingolimod (FTY720) in multiple sclerosis: two-year results of a phase II extension study. Neurology.

[6] Brinkmann V (2010). Fingolimod (FTY720): discovery and development of an oral drug to treat multiple sclerosis. Nat Rev Drug Discov.

[7] Chiba K (1998). FTY720, a novel immunosuppressant, induces sequestration of circulating mature lymphocytes by acceleration of lymphocyte homing in rats. I. FTY720 selectively decreases the number of circulating mature lymphocytes by acceleration of lymphocyte homing. J Immunol.

[8] Marsolais D, Rosen H (2009). Chemical modulators of sphingosine-1-phosphate receptors as barrier-oriented therapeutic molecules. Nat Rev Drug Discov.

[9] Stenovec M, Trkov S, Kreft M, Zorec R (2014). Alterations of calcium homoeostasis in cultured rat astrocytes evoked by bioactive sphingolipids. Acta Physiol (Oxf).

[10] Wu C (2013). Dual effects of daily FTY720 on human astrocytes *in vitro*: relevance for neuroinflammation. J Neuroinflammation.

[11] Cruz VT, Fonseca J (2014). Central effects of fingolimod. Rev Neurol.

[12] Foster CA (2007). Brain penetration of the oral immunomodulatory drug FTY720 and its phosphorylation in the central nervous system during experimental autoimmune encephalomyelitis: consequences for mode of action in multiple sclerosis. J Pharmacol Exp Ther.

[13] Czech B (2009). The immunomodulatory sphingosine 1-phosphate analog FTY720 reduces lesion size and improves neurological outcome in a mouse model of cerebral ischemia. Biochem Biophys Res Commun.

[14] Deogracias R (2012). Fingolimod, a sphingosine-1 phosphate receptor modulator, increases BDNF levels and improves symptoms of a mouse model of Rett syndrome. Proc Natl Acad Sci USA.

[15] Ruiz A (2014). Testing Aβ toxicity on primary CNS cultures using drug-screening microfluidic chips. Lab Chip.

[16] Takasugi N (2013). FTY720/fingolimod, a sphingosine analogue, reduces amyloid-β production in neurons. PLoS One.

[17] Vargas-Medrano J (2014). Novel FTY720-Based Compounds Stimulate Neurotrophin Expression and Phosphatase Activity in Dopaminergic Cells. ACS Med Chem Lett.

[18] Wei Y (2011). Fingolimod provides long-term protection in rodent models of cerebral ischemia. Ann Neurol.

[19] Balatoni B (2007). FTY720 sustains and restores neuronal function in the DA rat model of MOG-induced experimental autoimmune encephalomyelitis. Brain Res Bull.

[20] Cipriani R, Chara JC, Rodríguez-Antigüedad A, Matute C (2015). FTY720 attenuates excitotoxicity and neuroinflammation. J Neuroinflammation.

[21] Rossi S (2012). Oral fingolimod rescues the functional deficits of synapses in experimental autoimmune encephalomyelitis. Br J Pharmacol.

[22] Hait NC (2014). Active, phosphorylated fingolimod inhibits histone deacetylases and facilitates fear extinction memory. Nat Neurosci.

[23] Darios F (2009). Sphingosine facilitates SNARE complex assembly and activates synaptic vesicle exocytosis. Neuron.

[24] Flašker A, Jorgačevski J, Calejo AI, Kreft M, Zorec R (2013). Vesicle size determines unitary exocytic properties and their sensitivity to sphingosine. Mol Cell Endocrinol.

[25] García-Martínez V (2015). Sphingomyelin derivatives increase the frequency of microvesicle and granule fusion in chromaffin cells. Neuroscience.

[26] Rickman C, Meunier FA, Binz T, Davletov B (2004). High affinity interaction of syntaxin and SNAP-25 on the plasma membrane is abolished by botulinum toxin E. J Biol Chem.

[27] Hu K, Rickman C, Carroll J, Davletov B (2004). A common mechanism for the regulation of vesicular SNAREs on phospholipid membranes. Biochem J.

[28] Holt M, Riedel D, Stein A, Schuette C, Jahn R (2008). Synaptic vesicles are constitutively active fusion machines that function independently of Ca2+. Curr Biol.

[29] Connell E (2007). Mechanism of arachidonic acid action on syntaxin-Munc18. EMBO Rep.

[30] Davletov BA (1998). Vesicle exocytosis stimulated by alpha-latrotoxin is mediated by latrophilin and requires both external and stored Ca2+. EMBO J.

[31] Rupnik M, Zorec R (1995). Intracellular Cl- Modulates Ca2+-Induced Exocytosis from Rat Melanotrophs through Gtp-Binding Proteins. Pflugers Archiv-European Journal of Physiology.

[32] Rituper B (2013). High-resolution membrane capacitance measurements for the study of exocytosis and endocytosis. Nature Protocols.

[33] Gutierrez LM (1997). A peptide that mimics the C-terminal sequence of SNAP-25 inhibits secretory vesicle docking in chromaffin cells. J Biol Chem.

[34] Mosharov EV, Sulzer D (2005). Analysis of exocytotic events recorded by amperometry. Nat Methods.

[35] Kavalali ET, Klingauf J, Tsien RW (1999). Activity-dependent regulation of synaptic clustering in a hippocampal culture system. Proc Natl Acad Sci USA.

[36] Svennerholm L, Vanier MT, Månsson JE (1980). Krabbe disease: a galactosylsphingosine (psychosine) lipidosis. J Lipid Res.

[37] Neher E, Marty A (1982). Discrete changes of cell membrane capacitance observed under conditions of enhanced secretion in bovine adrenal chromaffin cells. Proc Natl Acad Sci USA.

[38] Lipovšek S, Janžekovič F, Leitinger G, Rupnik MS (2013). Rab3a ablation related changes in morphology of secretory vesicles in major endocrine pancreatic cells, pituitary melanotroph cells and adrenal gland chromaffin cells in mice. Gen Comp Endocrinol.

[39] Kreft, M., Jorgačevski, J., Vardjan, N. & Zorec, R. Unproductive Exocytosis. *J Neurochem* (2016). 10.1111/jnc.13561 26841731

[40] Vardjan N, Stenovec M, Jorgacevski J, Kreft M, Zorec R (2007). Subnanometer fusion pores in spontaneous exocytosis of peptidergic vesicles. J Neurosci.

[41] Wightman RM (1991). Temporally resolved catecholamine spikes correspond to single vesicle release from individual chromaffin cells. Proc Natl Acad Sci USA.

[42] Connell E, Scott P, Davletov B (2008). Real-time assay for monitoring membrane association of lipid-binding domains. Anal Biochem.

[43] Fragoso YD (2015). Persistent headache in patients with multiple sclerosis starting treatment with fingolimod. Headache.

[44] Ward MD, Jones DE, Goldman MD (2014). Overview and safety of fingolimod hydrochloride use in patients with multiple sclerosis. Expert Opin Drug Saf.

[45] Brosnan CF, Raine CS (2013). The astrocyte in multiple sclerosis revisited. Glia.

[46] Trkov S (2012). Fingolimod–a sphingosine-like molecule inhibits vesicle mobility and secretion in astrocytes. Glia.

[47] Safarian F, Khallaghi B, Ahmadiani A, Dargahi L (2015). Activation of S1P_1_ receptor regulates PI3K/Akt/FoxO3a pathway in response to oxidative stress in PC12 cells. J Mol Neurosci.

[48] Kavalali ET (2002). SNARE interactions in membrane trafficking: a perspective from mammalian central synapses. Bioessays.

[49] García-Martínez V (2013). Lipid metabolites enhance secretion acting on SNARE microdomains and altering the extent and kinetics of single release events in bovine adrenal chromaffin cells. PLoS One.

[50] Schröder M (2015). Subcellular distribution of FTY720 and FTY720-phosphate in immune cells - another aspect of Fingolimod action relevant for therapeutic application. Biol Chem.

[51] Jurado S (2013). LTP requires a unique postsynaptic SNARE fusion machinery. Neuron.

[52] Arendt KL (2015). Retinoic Acid and LTP Recruit Postsynaptic AMPA Receptors Using Distinct SNARE-Dependent Mechanisms. Neuron.

